# Introgressive hybridization and morphological transgression in the contact zone between two Mediterranean *Solea* species

**DOI:** 10.1002/ece3.2533

**Published:** 2017-02-03

**Authors:** Ahmed Souissi, Pierre‐Alexandre Gagnaire, François Bonhomme, Lilia Bahri‐Sfar

**Affiliations:** ^1^ Université de Tunis El ManarFaculté des Sciences de TunisUR 11ES08 Biologie integrative et écologie évolutive et fonctionnelle des milieu aquatiques, 2092TunisTunisie; ^2^ Université de MontpellierMontpellierFrance; ^3^ CNRS – Institut des Sciences de l'EvolutionUMR 5554 UM‐CNRS‐IRD‐EPHEStation Méditerranéenne de l'Environnement LittoralSèteFrance

**Keywords:** body shape, genetic–phenotypic correlation, introgressive hybridization, *Solea aegyptiaca*, *Solea senegalensis*, transgressive phenotypes

## Abstract

Hybrid zones provide natural experiments where new combinations of genotypes and phenotypes are produced. Studying the reshuffling of genotypes and remodeling of phenotypes in these zones is of particular interest to document the building of reproductive isolation and the possible emergence of transgressive phenotypes that can be a source of evolutionary novelties. Here, we specifically investigate the morphological variation patterns associated with introgressive hybridization between two species of sole, *Solea senegalensis* and *Solea aegyptiaca*. The relationship between genetic composition at nuclear loci and individual body shape variation was studied in four populations sampled across the hybrid zone located in northern Tunisia. A strong correlation between genetic and phenotypic variation was observed among all individuals but not within populations, including the two most admixed ones. Morphological convergence between parental species was observed close to the contact zone. Nevertheless, the samples taken closest to the hybrid zone also displayed deviant segregation of genotypes and phenotypes, as well as transgressive phenotypes. In these samples, deviant body shape variation could be partly attributed to a reduced condition index, and the distorted genetic composition was most likely due to missing allelic combinations. These results were interpreted as an indication of hybrid breakdown, which likely contributes to postmating reproductive isolation between the two species.

## 1 Introduction

Secondary contacts between closely related species, resulting from either natural processes or anthropogenic activity, often lead to the formation of hybrid zones in which populations with divergent genomes have the potential to exchange genes. These zones have been shown to act as semipermeable barriers to gene flow that selectively filter introgression by preventing genomic regions involved in reproductive isolation to be exchanged among species (Barton & Gale, 1993; Barton & Hewitt, 1985; Harrison, 1993; Harrison & Larson, 2014).

Depending on the traits considered, introgressive hybridization may lead to morphological convergence of parental populations if the underlying divergent genes behave neutrally and readily introgress upon secondary contact (Grant & Grant, 2002). A simple dilution of the parental phenotypic differences whereby hybrids display intermediate phenotypes compared to the two parental species is often observed in the populations located near the center of a hybrid zone (Mayr, 1963). Indeed, additive traits determined by quantitative traits loci (QTLs) with directional effects (with each QTL having an effect in one parental population but not in the other) should appear intermediate in hybrids compared to their parental populations (Albertson & Kocher, 2005; Rieseberg & Willis, 2007). Nevertheless, transgressive hybrid phenotypes that exceed the range of parental phenotypic variation have also been reported, in particular in the context of hybrid zones (Rieseberg, Archer, & Wayne, 1999; Rieseberg, Widmer, Arntz, & Burke, 2003). This phenomenon, referred to as transgressive segregation (Bell & Travis, 2005; Rieseberg, Archer et al., 1999; Stelkens, Schmid, Selz, & Seehausen, 2009), may be attributed on the contrary to traits encoded by QTLs with antagonistic effects in each parental population (Rieseberg, Archer et al., 1999).

Alternatively, morphological differences between species can persist despite genetic introgression if they are themselves involved in reproductive isolation, or if the genes that control them are tightly linked with genomic regions involved in reproductive isolation (Harrison & Larson, 2014). In this case, a link between morphological trait variation and individual fitness is expected (Arnold & Hodges, 1995). Some incompatible genotypic combinations may be eliminated, thereby preventing hybrids to reach the full range of phenotypic variation that could be potentially generated by recombining the parental genomes.

Disentangling the role of the different mechanisms that can be involved in producing morphological variation in hybrids remains fairly poorly studied in many taxa. Yet, it remains an important question for the study of hybrid zones (Gay, Crochet, Bell, & Lenormand, 2008), as it may help reveal the underlying architecture of reproductive isolation (Rieseberg, Whitton, & Gardner, 1999) as well as the origin of evolutionary novelties (Nichols et al., 2015; Parsons, Son, & Albertson, 2011).

Here, as a first step toward understanding the evolutionary constraints limiting gene flow across a species boundary, we explore body shape variation patterns along a natural hybrid zone transect between two species of sole, *Solea senegalensis* and *Solea aegyptiaca*. These two taxa, which geographical distributions partially overlap in the Mediterranean, are recognized as distinct sister species (Borsa & Quignard, 2001; Vachon, Chapleau, & Desoutter‐Meniger, 2008). Along the North African coast, *S. senegalensis* ranges from Senegal to Tunisia, and *S. aegyptiaca* occurs from Tunisia to Egypt. Few ambiguous morphological features have been described to distinguish them (Quéro, Desoutter, & Lagardère, 1986), and thus, these two taxa can be considered as cryptic species. Hence, the description of their spatial distributions relies primarily on genetics (Ouanes, Bahri‐Sfar, Ben Hassine, & Bonhomme, 2011; She, Autem, Kotulas, Pasteur, & Bonhomme, 1987). The two species come into contact in a 100‐km‐wide zone spreading from Bizerte in the north to Cap Bon in the south. This zone encompasses the Gulf of Tunis and the Bizerte Lagoon. Introgressive hybridization occurs and was evidenced using a few genetic markers in previous studies based on allozymes (She et al., 1987) and intron length polymorphisms (Ouanes et al., 2011). This phenomenon predominantly occurs in the large lagoon of Bizerte that appears to be the main habitat in which hybrids are found. It is however unclear whether this zone is stable or in the process of widening, because the average hybrid index observed in 2008 (41.2%; Ouanes et al., 2011) increased by three times the value measured in the same place twenty years earlier (16.1%) by She et al. (1987).

In this study, we propose to assess morphological variation patterns inside this zone and analyze the cosegregation of body shape and genetic variation. We use deviant segregation patterns at genetic markers as a way to capture possible mechanisms of selection against deleterious allelic combinations in introgressed genotypes. We also use body shape variation in admixed populations to evaluate the consequence of gene flow on the condition of introgressed individuals.

## 2 Materials and Methods

### 2.1 Sampling

**Figure 1:**
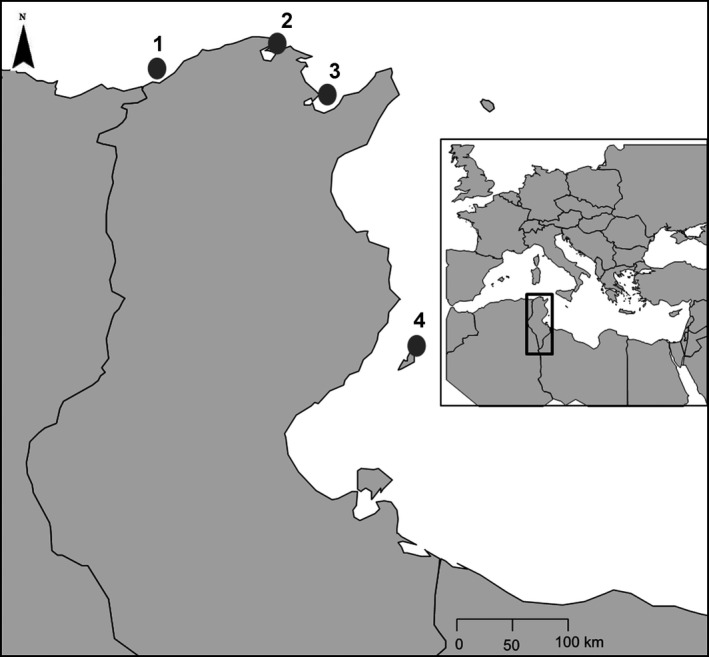
Sampling localities along the Tunisian coast. 1. Tabarka; 2. Bizerte Lagoon; 3. Gulf of Tunis; 4. Kerkennah Islands

We collected 88 adult individuals of *S. senegalensis* and *S. aegyptiaca* from four localities spanning as much as possible the hybrid zone between the two species along the Tunisian coast: Tabarka (*n* = 8), Bizerte Lagoon (*n* = 47), Gulf of Tunis (*n* = 15), and Kerkennah Islands (*n* = 14) (Figure 1). According to the previous study of Ouanes et al. (2011), these locations display variable amounts of genetic admixture. Tabarka contains *S. senegalensis* individuals introgressed by *ca*. 15% of *S. aegyptiaca* alleles. Conversely, on the opposite side, the Kerkennah sample contains mostly pure *S. aegyptiaca* individuals. These two locations constitute our peripheral samples. For the two inner samples, Bizerte Lagoon is considered as the nearest to the hybrid zone center and contains *S. senegalensis* genotypes admixed with *ca*. 41,2% of *S. aegyptiaca* alleles, while Gulf of Tunis contains *S. aegyptiaca* individuals introgressed by *ca*. 17% of *S. senegalensis* alleles. Individuals were collected from small fishing boats and transported immediately on ice to the laboratory for morphological and genetic experiments.

### 2.2 Genetic analysis

Whole genomic DNA (40 ng/μl) was extracted from fin clips using Qiagen DNeasy blood and tissue kit. Four EPIC loci (*GH2*,* Am2B‐2*,* CK6‐2,* and *Met1*) that were previously used to distinguish *S. senegalensis* and *S. aegyptiaca* outside the hybrid zone by Ouanes et al. (2011) were amplified by PCR under the same conditions. PCR products were separated by electrophoresis on a 1% acrylamide gel, and individual genotypes were subsequently scored with FMBIO II (Hitachi) using an internal size standard. Genotype scoring was performed twice by two different persons and then checked for consistency.

Discriminant correspondence analysis (DCA) based on group centroids as implemented in Genetix 4.05.2 (Belkhir, Borsa, Chikhi, Raufaste, & Bonhomme, 2004) was used to visualize the partitioning of genetic variation among individuals. This multivariate analysis is particularly well suited to describe the genetic composition of individuals in a gradient of admixture, as often encountered in hybrid zones. Genetic differentiation estimated by *F*_ST_ was assessed for each pair of samples and tested using 10,000 permutations in Genetix 4.05.2.

### 2.3 Morphometric analysis

**Figure 2:**
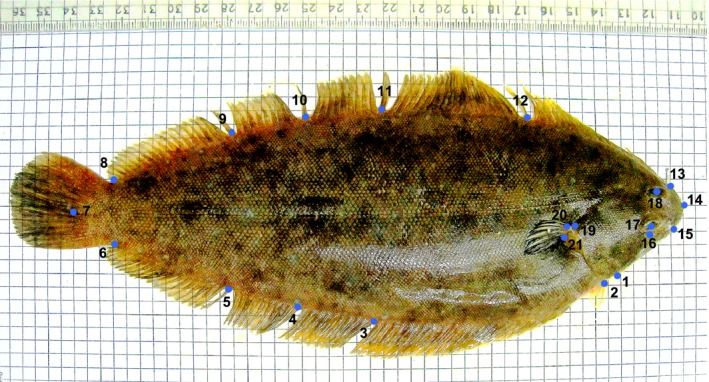
Positions of the digitized landmarks used for body shape analyses. 1. Insertion of operculum on the ventral profile; 2. origin of the pelvic fin; 3. insertion of the 40th anal fin ray (counted from posterior insertion of the anal fin); 4. insertion of the 30th anal fin ray (counted from posterior insertion of the anal fin); 5. insertion of the 20th anal fin ray (counted from posterior insertion of the anal fin); 6. ventral origin of the caudal fin; 7. caudal end of lateral line; 8. dorsal origin of the caudal fin; 9. insertion of the 20th dorsal fin ray (counted from posterior insertion of the dorsal fin); 10. insertion of the 30th dorsal fin ray (counted from posterior insertion of the dorsal fin); 11. insertion of the 40th dorsal fin ray (counted from posterior insertion of the dorsal fin); 12. insertion of the 60th dorsal fin ray (counted from posterior insertion of the dorsal fin); 13. anterior origin of the dorsal fin; 14. snout tip; 15. origin of the mouth (upper jaw); 16. end of the mouth (lower jaw); 17. center of the left ocular space; 18. center of the right ocular space; 19. end of operculum; 20. upper side of the pectoral fin; 21. lower side of the pectoral fin

Each individual was photographed on the eyed side with a Canon Digit Ixus 95 IS using a 35 mm f/2.8 lens with a fixed focal length. All images were digitized in TPSDig 2 (Rohlf, 2006) using 21 homolog landmarks to perform geometric morphometrics analysis based on two‐dimensional type I landmarks positioned according to clear homologous anatomical features (Figure 2). In order to remove nonshape variation, we performed a generalized Procrustes analysis using the R package *Geomorph* 2.1.7 (Adams & Otarola‐Castillo, 2013). This transformation consists in standardizing measures to control for differences in individual body size, by translating and rotating the configuration of landmarks to minimize the sum of squared distances between homologue landmarks (Zelditch, 2004). In order to test for remaining allometric effects following Procrustes transformation, we performed a multivariate regression of individual distance to the consensus shape against size using the procD.lm() function in *Geomorph*.

In order to focus on morphological variation between species, we then conducted a canonical discriminant analysis (CDA) to capture the multidimensional variation associated with body shape (Albrecht, 1980; Klingenberg, Barluenga, & Meyer, 2003; Zelditch, 2004). This method assesses the total amount of variation in body shape among groups of samples, expressed in a *n*‐dimensions space where *n* is the number of groups minus one. Transformation grids produced with the thin plate spline technique were used to visualize body shape changes among sampled populations. These analyses were performed with the R packages *Geomorph* (Adams & Otarola‐Castillo, 2013) and *Morpho* (Schlager, 2015).

### 2.4 Combining morphometric and genetic analysis

In order to evaluate the degree of similarity between genetic and shape variation among individuals, we used the procuste() function implemented in the *ade4* R package (Dray & Dufour, 2007). This method performs a simple Procrustes rotation of the hyperspaces resulting from the separate multivariate analyses to minimize the differences between the two clouds of homologous points and project them jointly in a new coordinate system (Digby & Kempton, 1987; Dray, Chessel, & Thioulouse, 2003). Finally, the significance of the concordance between genetic and morphometric data after Procrustes rotation (m²) was tested with the Procrustean randomization test (Protest) (Jackson, 1995; Peres‐Neto & Jackson, 2001).

## 3 Results

### 3.1 Genetic analyses

**Figure 3:**
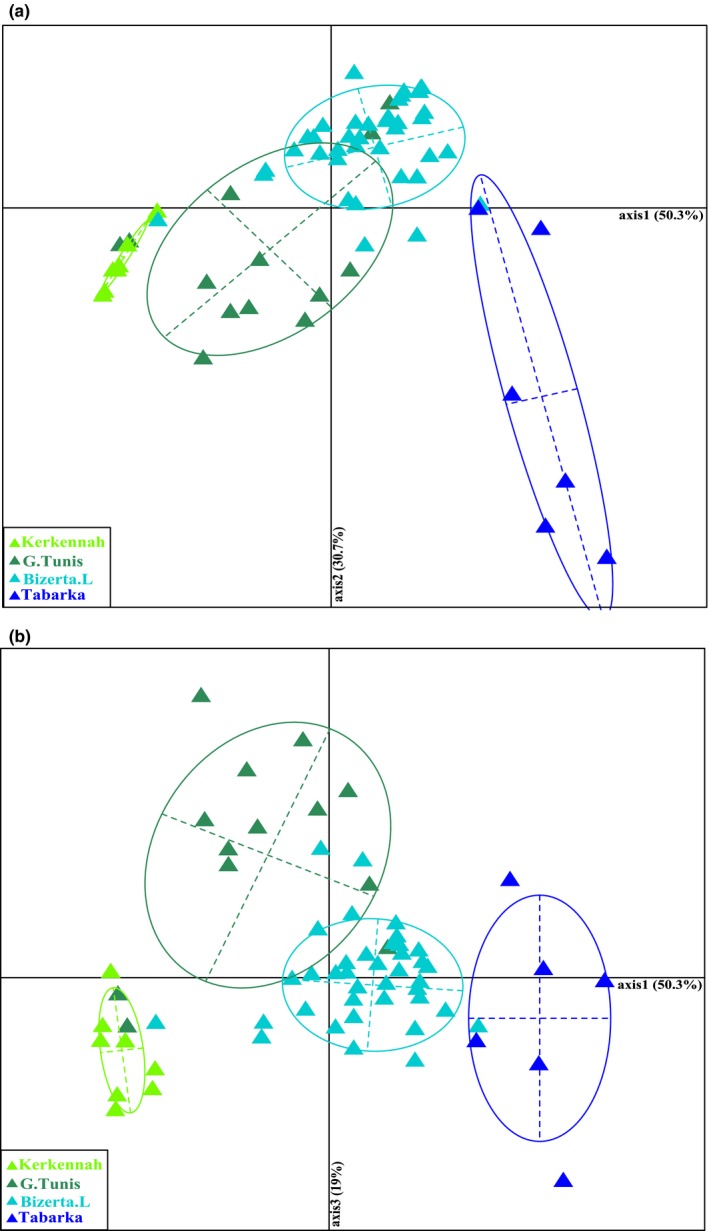
Discriminant correspondence analysis (DCA) on *Solea senegalensis* and *Solea aegyptiaca* based on Epic‐PCR Intronic markers (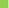 Kerkennah Islands, 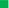 Gulf Of Tunis, 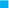 Bizerte Lagoon, and 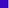 Tabarka). (a) Axis 1 and axis 2, (b) axis 1 and axis 3

As expected from previous studies, discriminant correspondence analysis (DCA) based on genetic data clearly separated the two samples from the periphery of the contact zone on the first axis, which explained most (50.3%) of the variation contained in the dataset. Tabarka specimens were positioned on the positive side of the first axis (*S. senegalensis* side of the hybrid zone), while specimens sampled in Kerkennah Islands were located on the negative part of this axis (*S. aegyptiaca* side of the hybrid zone) (Figure 3a). Fish collected in the inner samples stood in intermediate positions along this axis. Individuals from Bizerte Lagoon were more genetically similar to *S. senegalensis,* and Gulf of Tunis samples were more closely related to *S. aegyptiaca* (Table 1). The second axis of the DCA captured 30.7% of the total inertia, and essentially distinguished the Tabarka sample, revealing genetic differentiation between Tabarka and Bizerte samples on the *S. senegalensis* side of the hybrid zone. The third axis explained 19% of total genetic variation and mostly distinguished the Gulf of Tunis from the other three samples (Figure 3b).

**Table 1:** Genetic differentiation estimated by *F*_ST_ assessed for each pair of samples

*F*_ST_	Gulf of Tunis	Bizerte Lagoon	Tabarka
Kerkennah	0.358***	0.498***	0.732***
Gulf of Tunis		0.249***	0.384***
Bizerte Lagoon			0.328***

***p < .001.

### 3.2 Geometric morphometrics

**Figure 4:**
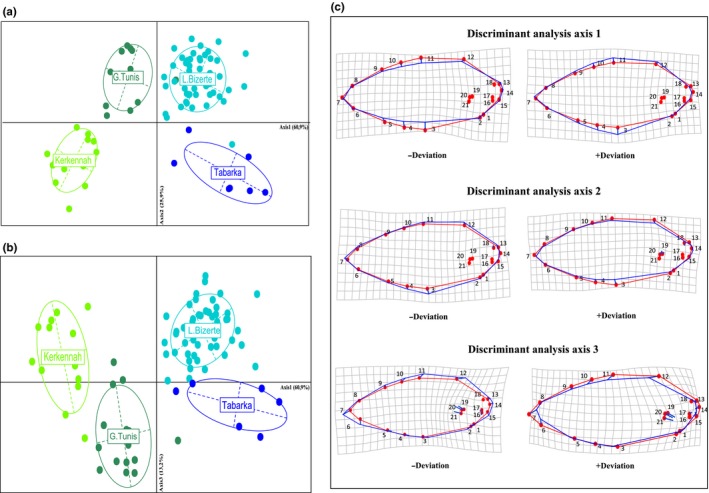
Canonical discriminant analysis (CDA) among *S. senegalensis* and *S. aegyptiaca* (Kerkennah Islands, Gulf of Tunis, Bizerte Lagoon, and Tabarka) based on geometric morphometrics (a) axis 1 and axis 2, (b) axis 1 and axis 3, (c) transformation grids associated to axis 1, axis 2, and 3. These transformation grids show the shape changes from the overall mean associated with each discriminant axis. These transformation grids show as blue hyphens the shape changes from the overall mean (red dots) associated with each discriminant axis. The scale factors for shape changes were set at the program's default values

Multivariate regression between Procrustes‐transformed shape coordinates and size showed no significant association (*p* = .12), indicating the absence of residual allometric effects after Procrustes transformation. The first axis of the CDA explained 60.9% of body shape variation and separated the four samples according to their geographic position relative to the center of the hybrid zone (Figure 4). The Tabarka sample located on the *S. senegalensis* side of the hybrid zone was projected in the positive part of axis 1, whereas Kerkennah Islands that is located on the *S. aegyptiaca* side of the hybrid zone was projected in the negative part of axis 1. The Gulf of Tunis and Bizerte samples occupied intermediate positions on each side of axis 1. The second and the third axes, which, respectively, explained 25.9% and 13.2% of shape variation, highlighted more complex patterns. On the one hand, axis 2 separated clearly the two inner samples (Bizerte Lagoon and Gulf of Tunis) from the two peripheral ones (Figure 4a). Hence, along this axis, the inner samples exhibited a morphological variance exceeding that of peripheral (and hence less introgressed) samples, which constitute a transgressive pattern. On the other hand, the third axis principally opposed the Gulf of Tunis and Bizerte samples that were the furthest apart along this axis (Figure 4b). This could be considered as another kind of transgressive pattern whereby the inner samples’ morphological variance also exceed that of peripheral ones, but in opposite directions.

The deformation grid obtained from the Procrustes superimposition showed that the principal body shape differences between species were associated with body height and were more specifically related to a dorsoventral expansion explained by landmarks 3, 4, 5, 10, 11, and 12. This differentiation follows a gradient along axis 1 in which the *S*. *senegalensis* individuals from Tabarka exhibit the largest body height, while the *S*. *aegyptiaca* individuals from Kerkennah have the lowest body height (Figure 4c). Deformation grid along axis 2 revealed a deformation component opposing the peripheral from the inner samples, whereby the latter have a less convex and more depressed abdomen than the former (landmark 3 and 4). Finally, shape variation along axis 3 was associated with the caudal (landmark 7), pectoral fin (landmark 19, 20, and 21), and head (landmark 14, 15, and 16) regions (Figure 4c).

### 3.3 Correlation between morphometry and genetics

**Figure 5:**
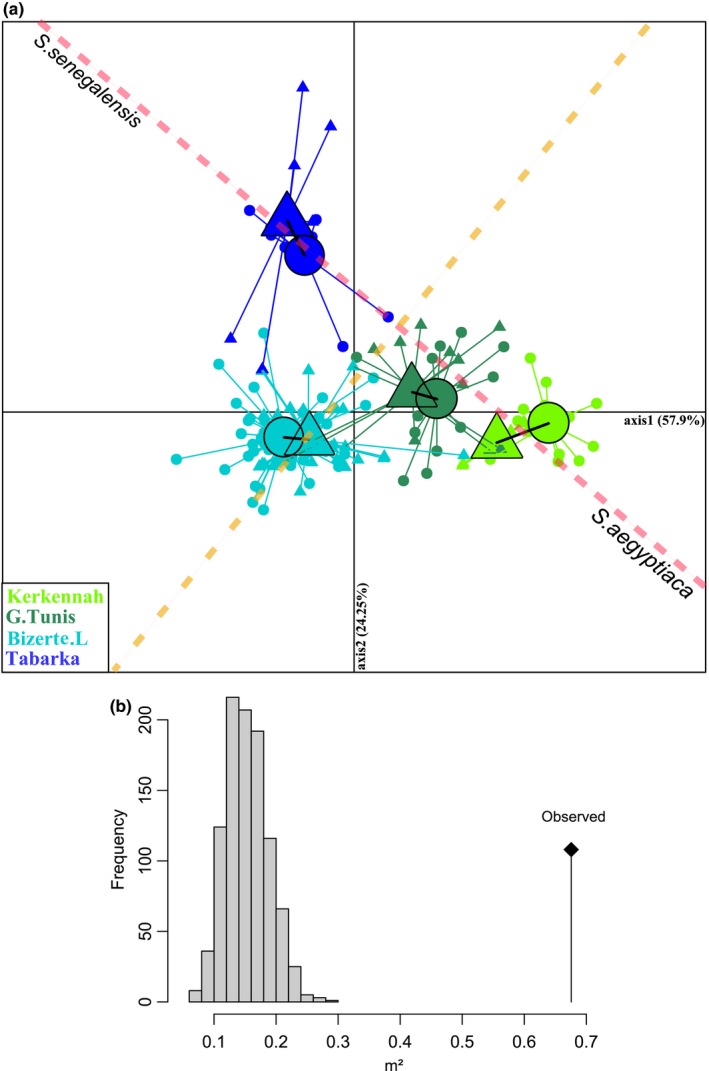
Plot of the genetic and morphometric data after Procrustes rotation (a) (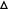: genetic data, 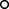: morphometric data; 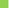 Kerkennah Islands, 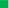 Gulf Of Tunis, 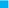 Bizerte Lagoon, and 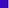 Tabarka). Correlation (m²) between the genetic and morphometric data after Procrustes rotation (b)

**Figure 6:**
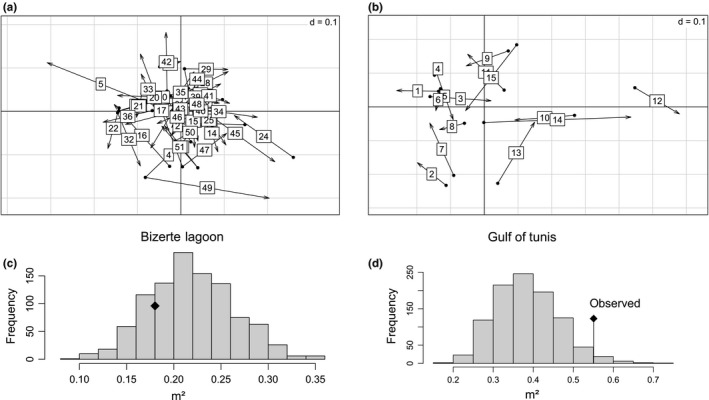
Plot of the genetic and morphometric data after Procrustes analysis performed within Bizerte Lagoon samples (a) and within Gulf of Tunis samples (b). The beginning of the arrow is the position of the genotype; the end of the arrow is the position of the phenotype. Correlation (m²) between the genetic and morphometric data after Procrustes rotation performed on Bizerte Lagoon (c) and Gulf of Tunis (d) samples

The Procrustes rotation of the bivariate configurations (i.e., axes 1 and 2) obtained from genetic and morphometric data analysis showed a high correlation between shape and genetic variation (m^2^ = 0.67, *p *< .001) (Figure 5b). In the new rotated plane, the main axis of morphogenetic differentiation between species was defined by a line connecting Tabarka and Kerkennah samples (red dashed line, Figure 5a). When projected on this new axis of differentiation between species, the two inner samples of Bizerte Lagoon and Gulf of Tunis occupied intermediate positions, as already observed with morphological and genetic analyses separately (Figures 3a and 4a). Moreover, a second axis of variation perpendicular to the first one (orange dashed line, Figure 5a) revealed a clearly shifted, position of the Bizerte sample, as to a lesser extent for the Gulf of Tunis sample. This significant correlation between genotype and phenotype and the intermediate positions for Bizerte Lagoon and Gulf of Tunis samples were still detected when the groups were not predefined in genetical and morphological analysis (m^2^ = 0.34, *p *< .001). Finally, performing the same Procrustes analysis within each samples separately revealed no significant correlation between genotype and phenotype variation (Bizerte Lagoon: m^2^ = 0.18, *p* = .8; Gulf of Tunis: m^2^ = 0.55, *p* = .028) (Figure 6).

## 4 Discussion

Our analysis of genetic variation across the hybrid zone between *S. senegalensis* and *S. aegyptiaca* is in good agreement with previous results by Ouanes et al. (2011). Here, using new samples, we confirm that Bizerte Lagoon and the adjacent Gulf of Tunis correspond to the area where the strongest admixture signal is found along the Tunisian coast. Moreover, these two locations are distributed on both sides of the hybrid zone, with Bizerte Lagoon being on the *S. senegalensis* side and Gulf of Tunis on the *S. aegyptiaca* side. As for genetic polymorphism, body shape variation also enabled us to discriminate easily between *S. senegalensis* and *S. aegyptiaca* morphotypes outside the zone. Moreover, morphological convergence was observed inside the zone where individuals from inner samples displayed intermediate morphologies compared to samples from parental populations (*i.e.,* in peripheral samples). The fact that morphological variation closely parallels genetic variation along the first axis in both analyses is expected under a simple dilution of additive genetic effects. Therefore, our data support that at least part of the geographic variation in body shape is explained by the neutral introgression of additive morphological traits along a gradient of admixture. Nevertheless, we found no significant correlation between morphological and genetic variation within samples, including those from the inner part of the hybrid zone. This was expected as most variation occurs between parental populations. Moreover, we only used a few genetic markers that are extremely unlikely to be linked with the loci controlling shape variation.

Our analysis reveals a different signal of variation along the second axis of the morphological space. On this axis, the two inner populations are clearly similar to each other while being differentiated from the parental outer samples which are themselves not differentiated along axis 2. This second pattern deviates from the previously mentioned mechanism of morphological convergence as it cannot be explained by additivity of the morphological QTLs alone. Thus, it suggests the existence of more complex epistatic and pleiotropic effects due to hybridization between divergent genomes. This could be due for instance to a mechanism of hybrid breakdown acting on different types of hybrid pedigree, as the population of Bizerte Lagoon is a *S. senegalensis* population introgressed by *S. aegyptiaca* alleles, whereas it is the opposite for the Gulf of Tunis. Phenotypic effects affecting all admixed genotypes in the same direction and contrasting with the absence of such effects in both parental populations could concern some fitness‐related trait reflecting the general performance of individuals, such as the condition factor. Interestingly, the deformation grid on the positive part of axis 2 shows a less convex shape of the ventral part in the inner samples which could reflect a lower condition of hybrid individuals compared to those from peripheral samples. In parallel, we also detected a signal of genetic distortion on axis 2 of the genetic correspondence analysis. This suggests the existence of preferential allelic combinations that cannot simply result from the neutral dilution of the parental genomes in introgressive crosses. Although we only used four neutral markers for genotyping, these selective effects can be likely captured due to genomewide association because of the existence of numerous genetic incompatibilities between highly differentiated (and sometimes coined “congealed”) genomes. The observed genetic distortions possibly reflect selective elimination of certain allelic combinations from the admixed populations, which may due to some form of hybrid breakdown. Such distortions in the wild closely reflect segregation distortions commonly observed in experimental crosses between divergent species (Casellas et al., 2012; Gagnaire, Normandeau, Pavey, & Bernatchez, 2013; Moyle & Graham, 2006). The depressed morphology of introgressed samples along axis 2 of the morphospace may thus constitute a phenotypic translation of this segregation load. Therefore, the morphogenetic correlation detected along axis 2 may reflect the fact that only hybrids combine at the same time admixed genotypes and lower condition.

Finally, a more classical pattern consistent with phenotypic transgression due to QTLs with antagonistic effects is evidenced along axis 3 of the morphospace. This effect is expected to occur in opposite directions on both sides of a hybrid zone as exemplified in Table 2. In one case, the predominant introgression of some *aegyptiaca* alleles inside the *senegalensis* background generates a positive transgression in the Bizerte sample and the opposite is true for the Gulf of Tunis sample.

**Table 2:** Hypothetical examples of transgressive segregation patterns involving six biallelic loci in two parental populations and their hybrid descendants

	Differentiated traits							
	Directional effects QTL				Antagonistic effects QTL			
	Sp1	Sp2	Introgressed Sp1	Introgressed Sp2	Sp1	Sp2	Introgressed Sp1	Introgressed Sp2
A	+1	0	+1	+1	+1	−1	+1	−1
B	+1	0	+1	0	+1	−1	+1	−1
C	+1	0	0	0	+1	−1	+1	−1
D	0	−1	0	−1	+1	−1	+1	−1
E	0	−1	−1	0	−1	+1	+1	+1
F	0	−1	0	0	−1	+1	−1	−1
∑	+3	−3	+1	0	+2	−2	+4	−4

To conclude, our study of the relationship between genetic composition and morphological shape highlighted mainly two key findings. First, when considering all four population samples, a significant correlation between genetic and phenotypic data was observed. This is expected because of the global linkage generated inside a tension zone maintained by equilibrium between counter‐selection of recombinant genotypes and gene flow from parental species. Second, deviant segregation of genotypes and phenotypes evidenced by transgressive positions of the most introgressed inner samples along axes 2 and 3 of the multivariate analyses could be interpreted both in terms of signature of hybrid breakdown and recombination of QTLs with antagonistic effects. Altogether, these results call for a genomewide assessment of the architecture of gene exchange between these two hybridizing species of sole. Such approach would be useful to specify what proportion of the genome can still be exchanged neutrally between species, and what are the fitness consequences of interspecific gene flow in recombinant genotypes.

## Conflict of Interest

None declared.
